# Dynamic Changes in Carbon and Nitrogen Storage and Sequestration of Alfalfa Pastureland in Different Planting Years Under Temperate Continental Arid Climate Conditions

**DOI:** 10.3390/plants14223432

**Published:** 2025-11-10

**Authors:** Xin Lu, Juan Qi, Xiangjun Meng, Junhu Su, Ximing Qi, Liyu Shen

**Affiliations:** 1 College of Grassland Science, Gansu Agricultural University, Lanzhou 730070, China; luxin61015@163.com (X.L.); sujh@gsau.edu.cn (J.S.); 13079335093@163.com (X.Q.); 15682838016@163.com (L.S.); 2 Key Laboratory of Grassland Ecosystem, Ministry of Education, Gansu Agricultural University, Lanzhou 730070, China; 3 Gansu Provincial Grassland Techniques Extension Station, Lanzhou 730010, China; mxj_712305@163.com

**Keywords:** arid regions, forage age, *Medicago sativa* L., productivity, utilization mode

## Abstract

Alfalfa (*Medicago sativa* L.), a drought-tolerant legume, significantly influences carbon and nitrogen cycling in arid and semi-arid regions. This study investigated carbon and nitrogen storage and sequestration dynamics in alfalfa pastureland cultivated for 2–7 years under temperate continental arid climate conditions (110–190 mm annual precipitation). Overall, the biomass, carbon and nitrogen sequestration in alfalfa pasture, and carbon and nitrogen storage and sequestration in soil exhibited a quadratic pattern with planting years. The above-ground biomass peaked at 19.28 t·hm^−2^, with carbon and nitrogen sequestration reaching the highest level at 10.18 t·hm^−2^ and 0.511 t·hm^−2^, respectively, in year 5. Both annual carbon and nitrogen sequestration of the below-ground vegetation exhibited an increase, reaching a peak before decreasing with planting year, and from Y3 to Y7, the sequestration values were consistently higher than those in Y2. Soil carbon and nitrogen sequestration peaked in year 3. Compared to the adjacent fallow lands, alfalfa pasturelands maintained positive soil carbon sequestration until year 6 but became negative (−8.03 t·hm^−2^) by year 7. From years 2–6, alfalfa pasture fixed carbon and nitrogen at comparable rates but returned disproportionately less carbon than nitrogen to the soil. To optimize sustainability, we recommend (1) rotating alfalfa after 6 years to prevent soil nutrient depletion and (2) applying carbon-rich fertilizers post-year 3 to balance nutrients and prolong productivity in arid climates.

## 1. Introduction

Global warming, driven by greenhouse gas emissions, makes climate action and sustainable development urgent worldwide priorities. The latest IPCC Sixth Assessment Report (2023) demonstrates that global temperatures have risen by 1.1 °C compared to pre-industrial levels over the past century, primarily due to fossil fuel combustion and unsustainable land use practices. This warming has triggered more frequent, intense and prolonged extreme precipitation events [1]. The sustainability of terrestrial carbon and nitrogen cycles in ecosystems is critical for preserving environmental resources [2,3]. Grasslands, covering approximately 25% of global land, are pivotal in carbon and nitrogen cycles. In China, grasslands constitute over 40% of the land area, 4 times its cropland and 3.6 times its forests [4]. They function as sources, sinks, and regulators, influencing regional and global climates [5,6]. Notably, 90% of grassland carbon and nitrogen is soil-stored, with soil organic carbon and total nitrogen levels determining soil quality and ecosystem productivity [7,8,9]. These cycles interact dynamically, driving complex ecosystem changes [10]. While expanding artificial grasslands enhances carbon and nitrogen sequestration, their efficiency varies with grass species and nutrient dynamics [11]. Therefore, accurate quantification of grassland carbon and nitrogen sequestration capacity is crucial for addressing climate change and ecological management.

Alfalfa (*Medicago sativa* L.), well-known for its high yield and nitrogen content, is a globally cultivated staple for artificial pasturelands in China. It bolsters food security and environmental sustainability through dual carbon sequestration and symbiotic nitrogen fixation [12,13]. However, prolonged cultivation degrades yields and soil health, causing soil desiccation and fertility loss [14,15,16,17]. Therefore, determining optimal planting duration is critical. Alfalfa’s growth cycle varies by cultivar, environment, and management. Planting duration is a primary determinant of soil carbon and nitrogen sequestration capacity in cultivated alfalfa stands. Studies show alfalfa fields increase soil organic carbon (SOC) by 10% and total nitrogen (TN) by 16% compared to croplands [18], with older fields (17 years) enhancing SOC in deeper layers [19]. Research has shown that in temperate semi-arid regions, SOC concentration and storage in alfalfa plantations peak during the 5th year of cultivation, with distinct surface accumulation patterns [20]. In contrast, in arid sandy soils, SOC concentrations reached their maximum earlier, peaking by the 4th year [21]. Cheng et al. [22] found that SOC concentration declined with cultivation duration. In plots cultivated for 5 and 8 years, SOC dropped to levels similar to surrounding fallow land (control), whereas 2-year cultivation maintained significantly higher SOC than control. These findings further underscore that the duration of alfalfa cultivation is a key factor influencing soil carbon sequestration efficiency. From the perspective of nitrogen fixation, alfalfa, through its symbiosis with rhizobia, could fix 148–177 kg of nitrogen per hectare annually, demonstrating significant nitrogen fixation capacity [20,23]. However, its nitrogen budget exhibited distinct stage-dependent characteristics. During the early years of cultivation (1–5 years), each harvest removed an average of 27 kg of nitrogen per ton of dry matter, while the soil nitrogen pool was in a state of net depletion. Only after the 5th year of alfalfa cultivation did the soil nitrogen content begin to accumulate [20]. Nevertheless, with the increase in cultivation duration, the intensity and stability of alfalfa’s nitrogen fixation ability still require further research and validation. In conclusion, determining the optimal cultivation duration for alfalfa and characterizing its carbon and nitrogen sequestration patterns are critically important for facilitating sustainable low-carbon agricultural development at the regional scale. To date, several research findings have been reported regarding the optimal cultivation duration of alfalfa. Regional studies suggest shorter cycles: ≤6 years in semi-humid areas [24], 3–5 years in arid areas, and 6–8 years in semi-arid zones [25]. It is evident that the optimal cultivation duration of alfalfa is significantly influenced by the climatic conditions of different regions, and the specific circumstances of each region still require further study.

The Hexi Corridor in northwest China features a typical temperate continental arid climate with abundant sunlight and thermal resources. Its saline–alkali soils challenge agriculture, including alfalfa pasture production. Alfalfa in this region is typically grown for over 7 years (up to 9+ years in some fields) before termination. The temporal dynamics of carbon and nitrogen storage and sequestration during the cultivation period, as well as their implications for ecosystem management, remain poorly understood. We hypothesized that the carbon and nitrogen sequestration potential of cultivated alfalfa in the Hexi Corridor follows a unimodal relationship with planting duration, increasing to a peak before declining, which defines an optimal cultivation period for maximizing ecological benefits. To test this hypothesis, we established a long-term field experiment with alfalfa stands aged 2 to 7 years, with the following objectives: (1) to quantify the annual carbon and nitrogen inputs and outputs, as well as sequestration; (2) to determine the impact of planting duration on soil carbon and nitrogen sequestration storages; and (3) to determine the optimal planting duration for maintaining high carbon and nitrogen sequestration capacity in alfalfa pasturelands in this region. The results provide essential data to optimize alfalfa pastureland management and support sustainable ecosystem development in the region.

## 2. Results

### 2.1. Above-Ground and Below-Ground Biomasses and Annual Carbon and Nitrogen Sequestration of Alfalfa Pasture

**Figure 1:**
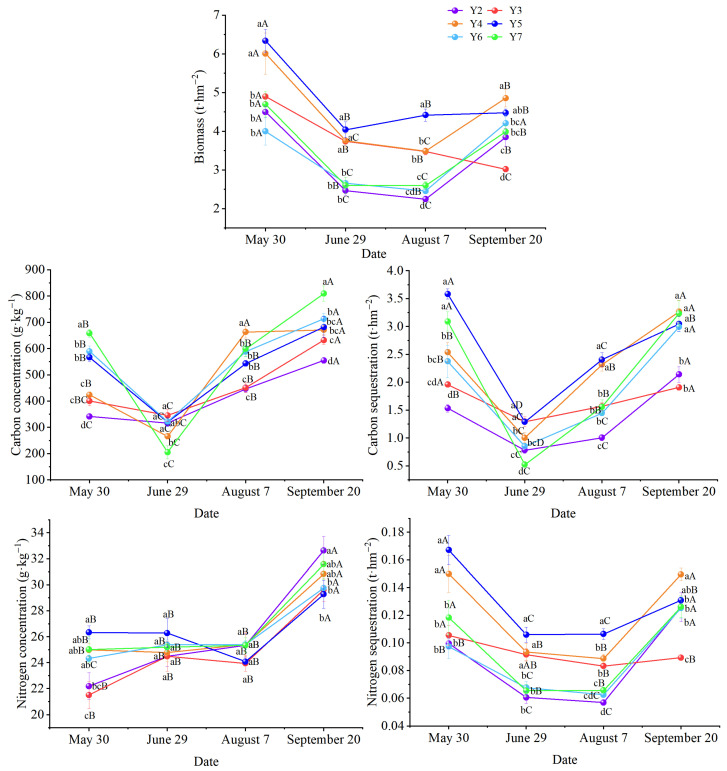
Variation in carbon and nitrogen storage of alfalfa shoots at different growth stages. Different uppercase letters denoted significant differences among different harvesting times within the same planting year, and different lowercase letters denoted significant differences across different planting years at the same harvesting time (*p* < 0.05).

As shown in Figure 1, alfalfa biomass and carbon–nitrogen sequestration exhibited dynamic seasonal trends. Across all stand ages, above-ground biomass, carbon concentration, and carbon–nitrogen sequestration consistently decreased early in the growing season before increasing later. In comparison, plant nitrogen concentration increased gradually during the early growth stages, followed by a rapid accumulation phase, peaking in September. The above-ground biomass of 4- and 5-year-old alfalfa stands was significantly higher than that of other age classes (*p* < 0.05).

The planting years of alfalfa significantly influenced biomass (*p* < 0.001), carbon concentration (*p* < 0.001), carbon sequestration (*p* < 0.001), and nitrogen sequestration (*p* < 0.001) in above-ground vegetation. However, they did not affect nitrogen concentration in alfalfa above-ground vegetation (*p* = 0.068). The planting years of alfalfa significantly influenced biomass, carbon concentration, carbon sequestration, nitrogen concentration, and nitrogen sequestration (*p* < 0.05) in below-ground vegetation (Table 1). The above-ground biomass of alfalfa pastureland increased to a peak before decreasing with the planting year, peaking in Y5 with a 47.5% increase compared to Y2. The carbon concentration increased linearly with the extension of the planting year. Meanwhile, the nitrogen concentration remained stable over the years, except for a lower value recorded in Y3. Both carbon and nitrogen sequestration peaked in Y5, showing increases of 87.8% and 48.9%, respectively, compared to Y2 (Table 1).

The below-ground biomass of alfalfa pastureland also peaked in Y5. Although no significant differences were observed among most planting years, the value in Y2 was significantly lower. (Table 1). The peak value in Y5 was 90% higher than that in Y2. The carbon concentration fluctuated over the 6 years without a clear trend. The nitrogen concentration ranged from 16.15 to 18.81 g·kg^−1^, peaking in Y4 (Table 1). Both annual carbon and nitrogen sequestration increased initially and then decreased with planting year, and from Y3 to Y7, the sequestration values showed no significant difference but were consistently higher than those in Y2 (Table 1). The peak sequestration values occurred in Y4, with increases of 105.9% for carbon and 55.9% for nitrogen compared to Y2.

### 2.2. Soil Moisture, Bulk Density, Carbon Concentration, and Nitrogen Concentration

**Figure 2:**
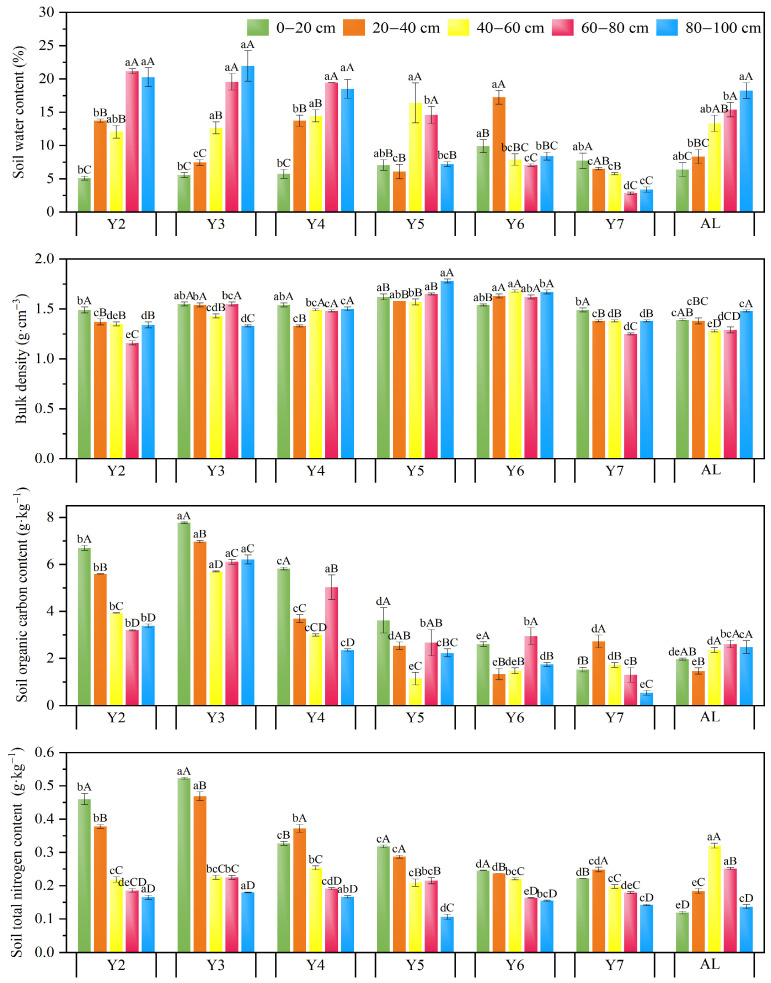
Soil water content (SW), bulk density (BD), soil organic carbon (SOC), and total nitrogen (TN) in alfalfa pastureland with different planting years in 2023. Different lowercase letters indicate significant differences between planting years within the same soil layer, and different uppercase letters indicate significant differences between different soil layers within the same planting year (*p* < 0.05).

As shown in Figure 2, soil water content varied significantly among the surface (0–20 cm), middle (40–60 cm), and deep (80–100 cm) layers across planting years. The soil water content in the 20–40 cm layer was significantly higher in Y2, Y4, and Y6 compared to the other planting years (*p* < 0.05). Soil bulk density changed dynamically with planting year, with minimal variation between layers. Except for Y6 and Y7, the SOC in the 0–20 cm layer was significantly higher than in other soil layers within the same year (*p* < 0.05). The average SOC concentration in the 0–100 cm soil was the highest in Y3 (43.64% higher than Y2) and lowest in Y7 (65.79% lower than Y2). As the planting year increased, TN concentration in each soil layer increased initially and then decreased with planting year. The average TN peaked in Y3. The TN concentrations in the 0–20 and 20–40 cm layers in Y3 were significantly higher than in other planting years (*p* < 0.05). Across all planting years, the TN concentration in the 0–40 cm layer was significantly higher than in the deeper soil layers (*p* < 0.05).

### 2.3. Soil Carbon and Nitrogen Storage

**Figure 3:**
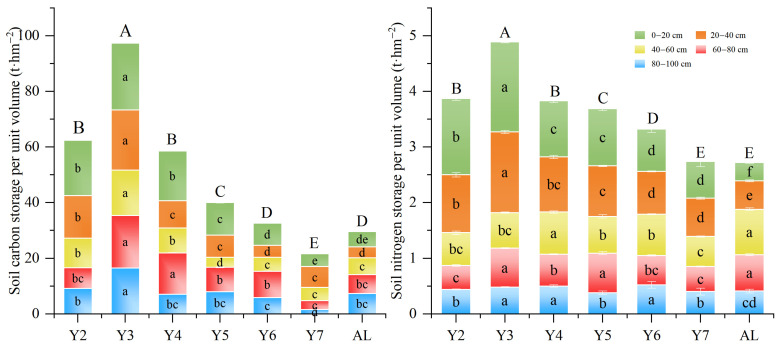
Soil carbon storage (SOCs) and total nitrogen storage (TNs) across soil layers in alfalfa pastureland in different planting years. Different lowercase and uppercase letters indicate significant differences among planting years for the same soil layer and for the total storage in the 0–100 cm profile, respectively (*p* < 0.05).

The soil organic carbon and nitrogen storage (t·hm^−2^) in alfalfa pastureland in different planting years are shown in Figure 3. The highest soil carbon storage (SOCs) was found in the 0–20 cm soil layer, followed by the 20–40 cm soil layer. The total SOCs in the 0–100 cm soil layer peaked in Y3, while there were no significant differences in SOCs between Y2 and Y4. Within the same planting year, TNs declined linearly with increasing soil depth. Across six planting years, TNs in the five soil layers, from the top to the bottom, contributed 28.3%, 25.9%, 18.0%, 15.4%, and 12.5%, respectively, to the total TNs in the soil. In the same soil layer, TNs was higher in Y3 and Y4 compared to the other planting years.

### 2.4. Soil Carbon and Nitrogen Sequestration Capacity

**Figure 4:**
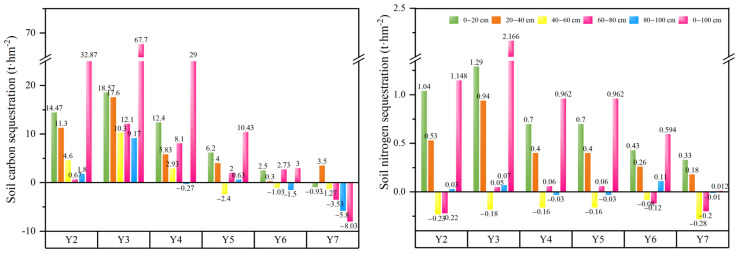
Soil carbon and nitrogen sequestration in various soil depths in alfalfa pastureland in different planting years in relation to the surrounding fallow lands.

As illustrated in Figure 4, the soil carbon and nitrogen sequestration in alfalfa pastureland relative to the surrounding fallow lands varied over different planting years. The carbon sequestration peaked in Y3 and reached its lowest point in Y7. The carbon sequestration in the 0–100 cm soil layer in Y7 was −8.03 t·hm^−2^, showing a significant carbon loss from alfalfa pastureland.

The nitrogen sequestration in soil followed a trend similar to carbon sequestration, reaching the peak value of 2.17 t·hm^−2^ in Y3 and the lowest point of 0.012 t·hm^−2^ in Y7. In contrast, the nitrogen sequestration in the 40–60 cm soil layer remained consistently negative across all years, indicating a net nitrogen loss compared to the fallow lands.

### 2.5. Total Fixed Carbon Amount and Total Fixed Nitrogen Amount in Alfalfa Pastureland

As shown in Table 2, the fixed carbon amount (t·hm^−2^) in above-ground vegetation, below-ground vegetation, soil, and their total in alfalfa pastureland generally increased initially and then decreased with planting years. The components peaked in different years: above-ground vegetation in Y5, below-ground vegetation in Y3, soil in Y2, and the total fixed carbon in Y2. The lowest values were recorded in Y2 for vegetation and in Y7 for both soil and the total. The fixed carbon in below-ground vegetation was 7% to 32% higher than in above-ground vegetation, except for similar values in Y5. Soil carbon contributed 55% to 86% of the total fixed carbon, with the percentage declining linearly after Y3.

The fixed nitrogen amount (t·hm^−2^) in above-ground vegetation, soil and the total fixed nitrogen in alfalfa pastureland increased initially and then decreased with planting years, with the peak value observed for above-ground vegetation in Y4 and for soil and the total in Y2. The fixed nitrogen in below-ground vegetation was low in Y2, reached the plateau value of 0.3 t·hm^−2^ from Y3 to Y5, and then slightly declined in Y6 and Y7. The total fixed nitrogen in above-ground vegetation was 23% to 101% higher than that in below-ground vegetation. Soil-fixed nitrogen accounted for 81% to 90% of the total.

**Figure 5:**
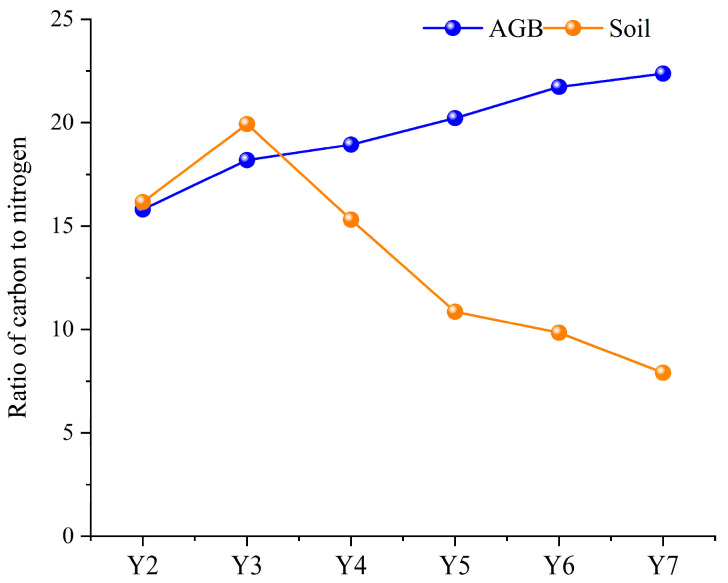
The ratio of the fixed carbon to nitrogen in above-ground biomass and soil in alfalfa pastureland in different planting years.

As shown in Figure 5, the ratio of carbon to nitrogen in above-ground vegetation exhibited a gradual increase from Y2 to Y7, rising from 15.8 in Y2 to 22.4 in Y7. In contrast, the ratio of carbon to nitrogen in the soil peaked in Y3 and subsequently declined steadily from 19.9 in year 3 to 7.9 in year 7.

### 2.6. Correlation Analysis Between Vegetation and Soil Characteristics

**Figure 6:**
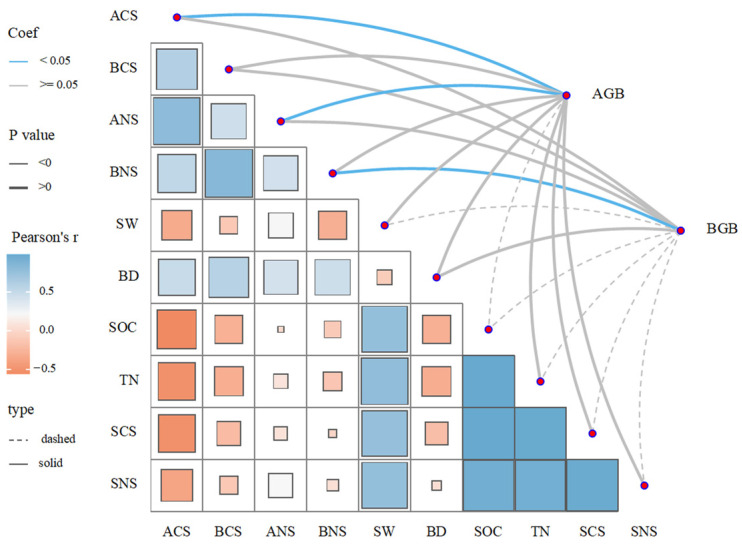
Relationship between soil carbon and nitrogen characteristics and vegetation characteristics. AGB: above-ground biomass; BGB: below-ground biomass; ACS: above-ground vegetation carbon sequestration; BCS: below-ground vegetation carbon sequestration; ANS: above-ground vegetation nitrogen sequestration; BNS: below-ground vegetation nitrogen sequestration; SW: soil water content; BD: bulk density; SOC: soil organic carbon; TN: soil total nitrogen; SCS: soil carbon sequestration; SNS: soil nitrogen sequestration. The same as below.

**Figure 7:**
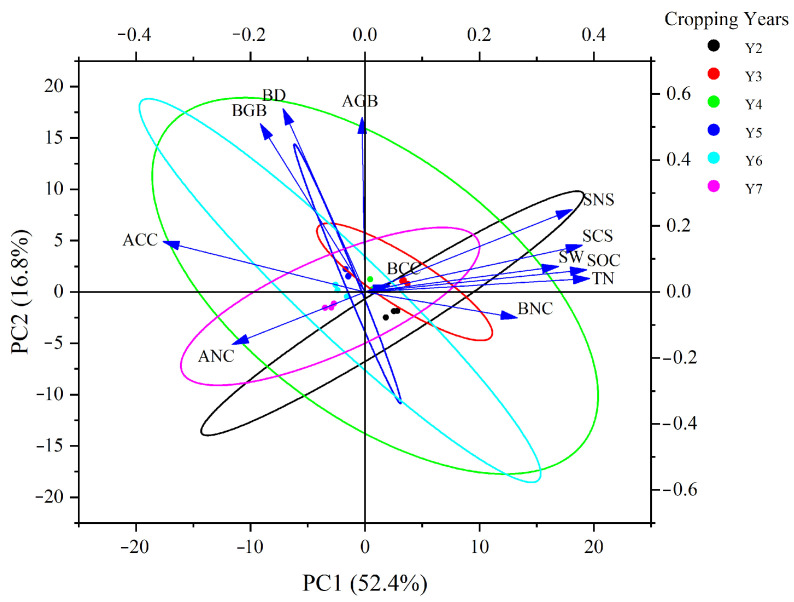
Principal component analysis of soil and vegetation characteristics across different alfalfa cultivation years. ACC: above-ground vegetation carbon concentration; BCC: below-ground vegetation carbon concentration; ANC: above-ground vegetation nitrogen concentration; BNC: below-ground vegetation nitrogen concentration.

Pearson’s correlation analysis revealed (Figure 6) above-ground vegetation carbon sequestration (ACS) was positively correlated with below-ground vegetation carbon sequestration (BCS), above-ground vegetation nitrogen sequestration (ANS), below-ground vegetation nitrogen sequestration (BNS), and BD but negatively correlated with SW, SOC, TN, soil carbon sequestration (SCS), and soil nitrogen sequestration (SNS). Principal component analysis (Figure 7) showed that the first principal component (PC1) explained over half of the total variance. AGB and BGB aligned closely with SOC, TN, SCS, and SNS, indicating that plant growth is strongly coupled with soil carbon and nitrogen accumulation. In contrast, soil BD showed a significant negative correlation with both productivity and soil fertility, indicating that soil porosity is a critical limiting factor for productivity and carbon–nitrogen sequestration.

## 3. Discussion

### 3.1. Above- and Below-Ground Biomass and Their Annual Carbon and Nitrogen Sequestration with Different Planting Ages

Alfalfa pasture, known for its high-yielding vegetation rich in nitrogen, serves as an ideal forage species for promoting livestock production in the local regions. Therefore, increasing above-ground biomass of the pasture is essential for sustaining the animal production system. In this study, we found a quadratic relationship between alfalfa pasture above-ground biomass and planting years, with the highest biomass produced in the 5th year of plantation, which was 147.5% of that produced in the 2nd year. The biomass subsequently declined in the 6th and 7th years, returning to the level comparable to the 2nd year. Our finding was supported by previous reports, which also found the maximum above-ground biomass of alfalfa in the 5th year of plantation in the semi-arid Loess Plateau regions [26,27]. However, under temperate continental climate conditions, alfalfa above-ground biomass peaked in the 3rd–4th years, with soil nutrient accumulation and alfalfa growth reaching an optimal balance during this period [28,29,30]. This variability in peak growth age across studies is likely attributed to differences in climate types and other environmental factors, such as precipitation (between 110 and 190 mm per annum in this study vs. 150–500 in these reports) and fertilizer management practices.

Notably, the carbon concentration in above-ground vegetation increased progressively with planting years, rising from 415 to 568 g·kg^−1^ (dry matter basis), suggesting that prolonged cultivation may enhance carbon sequestration in alfalfa pasture without compromising nitrogen content, a key factor for forage quality. Due to a combination of a quadratic pattern in above-ground biomass and a continuous increase in carbon concentration over planting years, carbon sequestration dynamics showed a quadratic trend over time. It peaked at 10.18 t·hm^−2^ in the 5th year, with the sequestration levels in the 6th–7th years remaining higher than those in the 2nd–3rd years. The carbon sequestration observed in this alfalfa pasture, except for the 2nd year, exceeds the 6 t·hm^−2^ reported for grasslands in China by Ni [31], highlighting alfalfa’s superior potential as a sustainable tool for agroecosystem carbon storage. Some studies have reported that alfalfa’s carbon concentration peaks in the 4th year of planting, which contrasts with our finding. This discrepancy may be attributed to variations in ecological conditions, as differences in water availability and temperature can influence both alfalfa biomass and carbon concentration [32]. The above-ground vegetation nitrogen sequestration followed a trend similar to that of the above-ground biomass, both peaking in the 5th year of alfalfa pasture, with an increase of 48.98% compared to the 2nd year.

Below-ground biomass, along with carbon and nitrogen sequestration, remained relatively stable over time, except for substantially lower values in year 2. This pattern suggested that the alfalfa root system reached its peak growth by year 3. This finding was aligned with a report on alfalfa pasture by Li et al. [33]. Notably, annual carbon sequestration in above-ground biomass was lower than in below-ground biomass, whereas annual nitrogen sequestration was higher in above-ground components. This divergence may reflect distinct biomass allocation strategies between above- and below-ground compartments during vegetation recovery, as well as the periodic harvesting of above-ground biomass [27].

### 3.2. Soil Water Content, Bulk Density, Carbon Contents and Sequestration in Alfalfa Pastureland in Different Planting Years

This study found that alfalfa pasture age affected soil water content and distribution across soil depth: 2–5-year-old plants lowered moisture in the shallow soil layers, whereas 6–7-year-old stands reduced moisture in the deeper soil layers. This pattern likely corresponds to root growth deepening with age, leading to increased water uptake from deeper soil layers. Research has demonstrated that soil moisture in alfalfa pasture is strongly dependent on local precipitation levels. However, across all climatic zones examined (arid, semi-arid, and semi-humid), alfalfa stands older than 6–7 years consistently exhibited significantly reduced soil moisture compared to 3-year-old stands, with increasing aridity observed as cultivation duration extended [34]. Researchers have also observed that alfalfa pastures in Australia consume at least 50 mm more soil moisture annually compared to annual forage grasses or crops, which can result in significant soil moisture depletion and even reduce groundwater recharge [35].

Dynamic changes in soil carbon influence the net balance of carbon, sustainable carbon cycles, and climate change. Alfalfa cultivation has been demonstrated to significantly enhance soil organic carbon sequestration potential, yet the time required to reach peak carbon sequestration effects exhibits notable regional variability [36]. For instance, in temperate semi-arid regions, Wu et al. [20] conducted an 8-year in situ observation study that revealed the SOC concentration and storage in alfalfa plantations peaked in the 5th year of cultivation, exhibiting pronounced surface accumulation characteristics. By contrast, in arid sandy transition zones, SOC concentration reached its maximum earlier, attaining peak levels by the 4th year of planting [21]. In this study, we found the average SOC concentration peaked in year 3 and thereafter declined sharply to the lowest value by year 7.

Importantly, alfalfa pastureland acted as a carbon sink from year 2 to year 6, whereas in year 7, it transitioned into a carbon source, exhibiting a significant carbon loss of 8.03 t·hm^−2^ compared to the surrounding fallow lands. The research by Cheng et al. [22] demonstrated that soil carbon sequestration showed a significant decreasing trend with extended cultivation duration. In plots cultivated for 5 and 8 years, the soil carbon sequestration had decreased to levels approaching those of surrounding fallow lands (controls), while in plots cultivated for only 2 years, the SOC concentration remained significantly higher than control levels. However, some studies have indicated that longer alfalfa cultivation periods were more conducive to soil carbon sequestration [36]. These discrepancies in research findings may be attributed to environmental factors such as climate types and soil characteristics across different study regions. These findings further confirm that alfalfa cultivation duration is a key factor influencing soil carbon sequestration efficiency.

Our study also revealed that SOC concentration decreased in the 0–60 cm soil layer but was generally higher in the 60–80 cm layer than in the 40–60 cm layer. This may be due to prolonged cultivation depleting topsoil (0–60 cm) nutrients, whereas the deeper 60–80 cm layer remained less exploited and retained more nutrients. Soil carbon sequestration followed a similar pattern to SOC concentration, peaking in year 3 and decreasing thereafter with an accelerating rate of carbon loss. Approximately half of the soil carbon was retained in the top 0–40 cm layer. Tai et al. [37] conducted soil sampling in 1–6-year-old alfalfa pastures (0–50 cm depth) and found that SOC concentration initially decreased, then rebounded with increasing soil depth.

### 3.3. Soil Nitrogen Contents and Sequestration in Alfalfa Pastureland in Different Planting Years

In terrestrial ecosystems, soil acts as the primary nitrogen reservoir, functioning dynamically as both a source and a sink through biogeochemical cycling [38,39]. Numerous studies have demonstrated that land use and management practices significantly influence soil nitrogen pool dynamics. The conversion of low-productivity croplands to alfalfa cultivation can enhance soil nitrogen content through the symbiotic nitrogen fixation system between alfalfa and rhizobia. However, the differential nitrogen uptake patterns across various alfalfa growth stages lead to substantial temporal variations in soil nitrogen levels during continuous cultivation [40,41,42].

More pronounced variations were observed in surface soils (0–40 cm), while deeper soil layers (40–60 cm) showed minimal changes across different cultivation years. Approximately 54% of total nitrogen storage was concentrated in the top 0–40 cm soil layer. Research findings from a published study showed a depth-dependent response of soil nitrogen content to prolonged cultivation, with significant variation in surface layers (0–30 cm) contrasting with stable subsoil levels (30–60 cm) across all growth stages [20]. This finding was consistent with our result and may be attributed to the fact that accumulation of large amounts of litter in the topsoil, along with favorable aeration and hydrothermal conditions, promotes nitrogen accumulation in surface soils [20,43,44].

From the perspective of soil nitrogen retention capacity, the maximum nitrogen sequestration (2.166 t·hm^−2^) occurred in year 3, followed by a steady decline to 0.012 t·hm^−2^ in year 7. While vertical stratification influenced nitrogen dynamics—with the top 0–40 cm soil layers acting as net sinks and the 40–60 cm layer functioning as a source—the total annual nitrogen sequestration in alfalfa pastureland consistently exceeded that of adjacent fallow lands. This demonstrates that alfalfa’s nitrogen sequestration sufficiently meets its metabolic requirement, reducing reliance on external nitrogen inputs.

### 3.4. Total Fixed Carbon and Nitrogen in Alfalfa Pastureland

Research on long-term carbon and nitrogen storage dynamics in artificial perennial pasturelands is relatively scarce [38]. Total fixed carbon peaked in year 3 (97.4 t·hm^−2^) and thereafter rapidly declined to 21.6 t·hm^−2^ by year 7, representing a 4.5-fold reduction from the maximum. Similarly, total fixed nitrogen decreased from its peak of 4.886 t·hm^−2^ in year 3 to 2.732 t·hm^−2^ in year 7, a 1.8-fold decline. These results indicate that, after the 3rd year, that is, two years before the peak biomass, the input of carbon and nitrogen into the soil begins to decrease in alfalfa pastures. Likely, slow growth of alfalfa pasture during the first few years facilitates soil nutrient accumulation, which subsequently supports rapid biomass growth in later years.

We noted that the ratio of the fixed carbon to nitrogen in soil continued to decline markedly from 19.9 in year 3 to 7.9 in year 7 (a 2.5-fold decrease), while the ratio in above-ground biomass increased modestly from 18.1 to 22.4 (1.2-fold), as illustrated in Figure 5. This divergence suggests that while alfalfa pasture fixes carbon and nitrogen at comparable rates throughout its lifespan, it returns disproportionately less carbon relative to nitrogen to the soil. As a legume, alfalfa introduces external nitrogen via symbiotic nitrogen fixation but lacks mechanisms to supplement soil beyond root exudates and litter decomposition. Consequently, soil carbon depletion outpaces nitrogen reduction over time, particularly in arid ecosystems where microbial mineralization rates are elevated [45].

These findings confirm that alfalfa cultivation enhanced soil carbon and nitrogen sequestration compared to the adjacent fallow lands, thereby accelerating ecosystem nutrient cycling. In the study region, an arid zone with over 110 mm annual precipitation, 3- and 4-year-old alfalfa pasture developed mature root systems that optimized nutrient uptake and exudate production [46,47]. However, by years 6–7, soil organic carbon and total nitrogen content declined markedly, indicating net nutrient depletion. This depletion results from a progressive shift in plant–soil interactions. In the first 3–5 years, vigorous alfalfa growth and root exudates promote SOC accumulation. As plants age, however, physiological senescence sets in, reducing root activity and organic matter input. Continued nutrient uptake leads to a net nutrient export; this effect is particularly pronounced when above-ground biomass is harvested and removed. Together with arid-induced water stress and C:N imbalances, these factors collectively deplete soil carbon and nitrogen pools [48].

To mitigate this imbalance and sustain productivity, we recommend targeted carbon supplementation (e.g., compost or biochar) beginning in the third year. Alternatively, rotating alfalfa after 6 years with legume–grass mixtures or gramineous species such as *Bromus inermis* Layss., which contributes high-carbon residues that enhance SOC and improve soil physical structure [49]. In continuous alfalfa systems, such interventions may extend the high-yield phase by an additional 2–3 years.

## 4. Materials and Methods

### 4.1. Study Site

**Figure 8:**
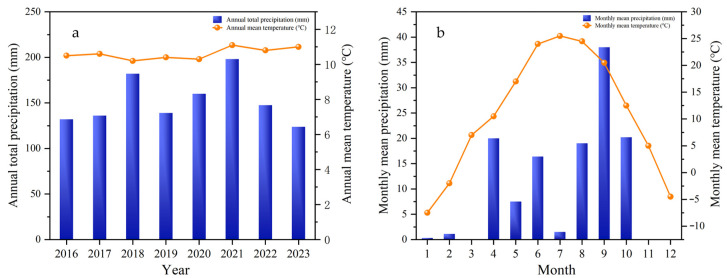
(**a**) Averaged annual temperature and precipitation in the study area from 2016 to 2023. (**b**) Monthly temperature and precipitation data in 2023.

The study area is located in Minqin County, Gansu Province, within the Hexi Desert Irrigation Area (38°13′ N, 102°42′ E; elevation: 1412 m). It is characterized by a typical temperate continental arid climate, featuring abundant solar radiation, distinct seasons, and a large diurnal temperature range. The mean annual temperature is 7–8 °C, with approximately 110 mm of annual precipitation occurring predominantly in summer and autumn. The region experiences high annual evaporation (2483 mm) and has a mean relative humidity of 44%. The distributions of precipitation and temperature are presented in Figure 8. The soil has sandy or sandy–loam texture with low soil organic matter content. The basic soil properties at the experimental site were as follows: bulk density: 1.36 g·cm^−3^; soil pH: 7.59; soil organic carbon: 4.95 g·kg^−1^; total soil nitrogen: 0.26 g·kg^−1^.

### 4.2. Experimental Design

In October 2023, based on the space-for-time substitution method [50,51,52], alfalfa sample plots with different planting years (2-year-old (Y2), 3-year-old (Y3), 4-year-old (Y4), 5-year-old (Y5), 6-year-old (Y6), and 7-year-old (Y7)) were selected from areas with similar terrain conditions and consistent management measures. Here, “planting year” refers to the number of consecutive growing cycles from the year of initial establishment (sowing year) to the sampling year for alfalfa. Experimental plots were established annually from 2017 to 2022, representing stands aged 2 to 7 years, respectively, with three replicate plots for each establishment year. The alfalfa variety planted was WL363, sown in rows with 20 cm spacing, a sowing depth of 2 cm, and a seeding rate of 37.5 kg·hm^−2^. At the time of alfalfa planting each year, 150 kg·hm^−2^ of diammonium phosphate was applied as a basal fertilizer. Since the beginning of the experiment, field management measures have been generally consistent across all plots. All alfalfa pastures received drip irrigation biweekly regardless of cultivation years. The irrigation process was controlled by an automatic timer, ensuring continuous water delivery to each emitter and consistent water volume across all experimental plots. Pesticides were sprayed, and manual weeding was performed in a timely manner. Pest conditions were monitored closely, and during periods of high pest and disease incidence, Imidacloprid 10% EC (Hebei Saireed Chemical Co., Ltd., Shijiazhuang, China) was diluted with clean water at a ratio of 1:2000. Applications were made using a drone, with the total spray volume controlled between 450 L·hm^−2^ to ensure uniformity across all treatments [53].

### 4.3. Plant Collection and Analysis

To capture the dynamic accumulation process of carbon and nitrogen stocks in alfalfa over a complete growing season. The above-ground biomass of alfalfa was measured during four harvests (30 May, 29 June, 7 August, and 20 September 2023). For above-ground biomass, plants were randomly collected from three 1 m × 1 m quadrats per plot at each harvest. The fresh weight of the grass was recorded, and a 1.5 kg sample from each quadrat was taken and transported to the laboratory. After drying at 105 °C for 30 min, followed by oven-drying at 65 °C for two days to a constant weight, the dry weight was measured to calculate the above-ground biomass per unit area. Following the final harvest, roots from the 0 to 100 cm soil layer in three additional 1 m^2^ quadrats were excavated, washed, and dried using the same protocol to measure below-ground biomass. Organic carbon content measurement in vegetation was determined using the potassium dichromate–external heating method, and total nitrogen content was measured using the sulfuric acid catalyst digestion–Kjeldahl method [54].

### 4.4. Soil Sampling and Analysis

In October 2023, soil samples were collected from each experimental plot for comprehensive physical and chemical properties. Three 1 m × 1 m quadrats were randomly selected per plot, from which triplicate soil cores were extracted across five distinct depth intervals (0–20, 20–40, 40–60, 60–80, and 80–100 cm) using the profile method [11]. All collected samples were homogenized and sequentially sieved through 1 mm and 0.25 mm stainless steel mesh.

Physical property assessments included [54] (1) soil bulk density determination via the ring knife method, with triplicate measurements conducted for each depth interval, and (2) soil water content determination using the drying method at 130 °C. Chemical analyses encompassed [54] (1) organic carbon content measurement in soil was determined using the potassium dichromate–external heating method, and (2) total nitrogen content measurement using the sulfuric acid catalyst digestion–Kjeldahl method. All analytical procedures were performed in triplicate to ensure methodological rigor and data reproducibility.

### 4.5. Data Calculation and Statistical Analysis

#### 4.5.1. Carbon and Nitrogen Storage

Vegetation carbon and nitrogen storage were calculated based on vegetation biomass and its carbon/nitrogen concentrations on a dry matter basis. Vegetation organic carbon storage (*GBCD_i_*, t·hm^−2^) and nitrogen storage (*GBND_i_*, t·hm^−2^) were calculated as follows [55]:

(1) $${GBCD}_{i}={NPP}_{i}\times C_{i}\times 0.001$$

(2) $${GBND}_{i}={NPP}_{i}\times N_{i}\times 0.001$$

In Formulas (1) and (2), *GBCD_i_* represents the organic carbon storage (t·hm^−2^) of vegetation in the *i*th plot; *GBND_i_* denotes the total nitrogen storage (t·hm^−2^) of vegetation in the *i*th plot; *NPP_i_* refers to the net primary production (dry weight) (t·hm^−2^) of vegetation in the *i*th plot; *C_i_* represents the organic carbon concentration (g·kg^−1^) of the vegetation in the *i*th plot, and *N_i_* represents the total nitrogen concentration (g·kg^−1^) of the vegetation in the *i*th plot. The factor of 0.001 was used to maintain unit consistency by converting *C_i_* and *N_i_* from g·kg^−1^ to t·hm^−2^, thereby yielding storage values in this unit. The total organic carbon storage and nitrogen storage of alfalfa pastureland were the sum of the organic carbon and total nitrogen storage of each vegetation cutting for the year, with the unit being t·hm^−2^.

The calculation formulas of soil organic carbon storage (*SOCs*, t·hm^−2^) and total nitrogen storage (*TNs*, t·hm^−2^) were as follows [56]:

(3) $$SOCs=H_{i}\times B_{i}\times SOC\times 0.1$$

(4) $$TNs=H_{i}\times B_{i}\times TN\times 0.1$$

In Formulas (3) and (4), *H_i_* represents the thickness (cm) of the *i*th soil layer, *B_i_* represents the bulk density (g·cm^−3^) of the *i*th soil layer, *SOC* refers to the soil organic carbon concentration (g·kg^−1^), and *TN* indicates the total nitrogen concentration (g·kg^−1^). The *i* represents the specific soil layer. The factor of 0.1 was a unit conversion factor used to integrate the units of cm, g·cm^−3^, and g·kg^−1^ into the final unit of t·hm^−2^. The annual total organic carbon and nitrogen storage in the 0–100 cm soil layer was the sum of the soil organic carbon and total nitrogen storage across all individual soil layers.

#### 4.5.2. Soil Carbon and Nitrogen Sequestration Capacity of Alfalfa Pastureland

This study used the soil organic carbon and nitrogen storage of the surrounding fallow lands (AL) as the baseline. The vegetation on the fallow land consisted of a few weeds, and no crops were cultivated. The soil sample collection method for the fallow land was consistent with the method used for the alfalfa soil samples of different years. The soil carbon and nitrogen sequestration capacity of the alfalfa pasturelands was defined as the difference between the soil organic carbon and nitrogen storage of the alfalfa pasturelands and that of the fallow lands over different years. A positive difference indicated that the establishment of the alfalfa pasturelands results in carbon and nitrogen sequestration, while a negative difference indicated carbon and nitrogen loss.

#### 4.5.3. Total Fixed Carbon Amount and Total Fixed Nitrogen Amount of Alfalfa Pastureland

The fixed carbon amount in alfalfa pasturelands was calculated as the sum of carbon sequestration in above-ground vegetation, below-ground vegetation and soil. Similarly, the total fixed nitrogen was determined by summing the nitrogen sequestration in above-ground vegetation, below-ground vegetation and soil.

### 4.6. Statistical Analysis

Statistical analyses were conducted using SPSS (version 26.0; IBM Corporation, Armonk, NY, USA). One-way analysis of variance (ANOVA) was used to assess differences among planting years, followed by Duncan’s multiple range test for post hoc comparisons. *p*-values less than 0.05 were considered statistically significant. Data were presented as mean ± standard error. To examine the relationship between changes in vegetation and soil physicochemical properties and those in AGB and BGB, we used the “linkET” package to perform Mantel test correlation analysis. Principal component analysis (PCA) was carried out in Origin Pro on standardized vegetation and soil properties to evaluate the integrated effect of planting duration. Figures were generated using Origin Pro software (Version 2021, Origin Lab Corporation, Northampton, MA, USA) and R 4.0.3 (R Development Core Team, 2020).

## 5. Conclusions

Our findings collectively demonstrate that in a temperate continental arid climate, the carbon and nitrogen sequestration dynamics in alfalfa pasturelands follow a clear temporal sequence. The soil C and N pools peaked earlier (year 3) than above-ground biomass and vegetation sequestration (year 5). This asynchrony indicates that relying solely on above-ground productivity risks soil overexploitation. We therefore propose using the peak in soil C-N indicators (year 3) as an early-warning signal for management intervention. Compared to the adjacent fallow lands, alfalfa pastureland maintained positive soil carbon sequestration until the 6th year but turned into a net carbon source by the 7th year. From years 2 to 6, alfalfa pasture fixed carbon and nitrogen at comparable rates but returned disproportionately less carbon than nitrogen to the soil. Soil physical properties, particularly bulk density, were identified as major limiting factors for both biomass accumulation and soil carbon–nitrogen sequestration. Therefore, rotating alfalfa pastureland after 6 years is proposed to prevent soil nutrient depletion. Applying carbon-rich fertilizers after 3 years of cultivation could assist in balancing soil nutrients and extending the productive lifespan of alfalfa pastureland.

## Figures and Tables

**Table 1:** Carbon and nitrogen sequestration of above-ground and below-ground vegetation of alfalfa pastureland with different planting ages.

Planting Years	Biomass (t·hm^−2^)	Carbon Concentration (g·kg^−1^)	Nitrogen Concentration (g·kg^−1^)	Carbon Sequestration (t·hm^−2^)	Nitrogen Sequestration (t·hm^−2^)
Above-ground vegetation					
Y2	13.07 ± 0.20 c	415.0 ± 13.85 e	26.17 ± 2.64 a	5.42 ± 0.09 e	0.343 ± 0.01 b
Y3	15.15 ± 0.05 b	457.5 ± 24.28 d	24.90 ± 0.63 b	6.93 ± 0.18 d	0.370 ± 0.00 b
Y4	18.13 ± 0.94 a	506.1 ± 6.48 c	26.50 ± 1.96 a	9.20 ± 0.44 b	0.482 ± 0.03 a
Y5	19.28 ± 0.20 a	528.2 ± 23.06 bc	26.50 ± 1.76 a	10.18 ± 0.27 a	0.511 ± 0.01 a
Y6	13.33 ± 0.36 c	553.4 ± 23.11 ab	26.22 ± 0.17 a	7.36 ± 0.28 c	0.353 ± 0.01 b
Y7	13.89 ± 0.44 c	568.3 ± 48.29 a	26.79 ± 0.98 a	7.89 ± 0.15 bc	0.376 ± 0.01 b
SEM	0.2318	5.2038	0.1996	0.1304	0.00
*p* value	<0.001	<0.001	0.068	<0.001	<0.001
Below-ground vegetation					
Y2	9.56 ± 0.27 B	608.5 ± 41.6 B	17.92 ± 0.13 AB	5.80 ± 0.29 B	0.171 ± 0.00 B
Y3	16.68 ± 0.46 A	533.9 ± 46.9 B	18.11 ± 0.10 AB	8.86 ± 0.95 A	0.302 ± 0.01 A
Y4	16.37 ± 1.61 A	727.0 ± 58.5 A	18.81 ± 0.21 A	11.94 ± 1.78 A	0.307 ± 0.03 A
Y5	18.22 ± 0.82 A	564.0 ± 27.4 B	16.69 ± 0.62 B	10.26 ± 0.59 A	0.304 ± 0.02 A
Y6	16.27 ± 2.32 A	636.0 ± 75.6 AB	16.15 ± 0.42 B	10.06 ± 0.93 A	0.263 ± 0.03 A
Y7	16.89 ± 0.16 A	535.8 ± 20.4 B	17.17 ± 0.46 B	9.04 ± 0.28 A	0.290 ± 0.01 A
SEM	0.6116	17.3581	0.1871	0.4763	0.0026
*p* value	0.004	0.015	0.003	0.014	0.003

Note: Different lowercase letters indicate significant differences in above-ground vegetation indicators across planting ages (*p* < 0.05), while values followed by different uppercase letters indicate significant differences in below-ground vegetation indicators across planting ages (*p* < 0.05). Data are presented as mean ± SE.

**Table 2:** Total fixed carbon amount and total fixed nitrogen amount in alfalfa pastureland with different planting years.

Planting Years	Fixed Carbon Amount (t·hm^−2^)				Fixed Nitrogen Amount (t·hm^−2^)			
	Above-Ground Vegetation	Below-Ground Vegetation	Soil	Total	Above-Ground Vegetation	Below-Ground Vegetation	Soil	Total
Y2	5.42	5.80	62.5	73.72	0.343	0.171	3.868	4.314
Y3	6.73	8.86	97.4	112.99	0.370	0.302	4.886	5.472
Y4	9.13	11.94	58.5	79.57	0.482	0.307	3.820	4.689
Y5	10.33	10.26	40.0	60.59	0.511	0.304	3.682	4.515
Y6	7.67	10.06	32.6	50.33	0.353	0.261	3.314	3.914
Y7	8.41	9.04	21.6	39.05	0.376	0.290	2.732	3.366

## Data Availability

All the required data are uploaded as Supplementary Materials.

## Supplementary Materials

The following supporting information can be downloaded at https://www.mdpi.com/article/10.3390/plants14223432/s1, Table S1: Inter-annual (2016–2023) and monthly (2023) data for the average annual temperature and precipitation in the study area; Table S2: Raw data on carbon and nitrogen sequestration in above-ground and below-ground vegetation of alfalfa pastureland with different planting ages; Table S3: Raw data on soil physical and chemical properties: bulk density, moisture content, and carbon/nitrogen concentrations and stocks as affected by soil layer and planting Year.
